# Anti-repulsive Guidance Molecule C (RGMc) Antibodies Increases Serum Iron in Rats and Cynomolgus Monkeys by Hepcidin Downregulation

**DOI:** 10.1208/s12248-015-9770-4

**Published:** 2015-04-22

**Authors:** Preethne Böser, Dietmar Seemann, Michael J. Liguori, Leimin Fan, Lili Huang, Mathias Hafner, Andreas Popp, Bernhard K. Mueller

**Affiliations:** AbbVie Deutschland GmbH & Co. KG, Knollstrasse, 67061 Ludwigshafen, Germany; AbbVie Inc, North Chicago, Illinois USA; AbbVie Bioresearch Center, Worcester, Massachusetts USA; Institute for Medical Technology, Heidelberg University and University of Applied Sciences, Mannheim, Germany

**Keywords:** ABT-207, h5F9-AM8, hepcidin, PK/PD analysis, safety assessment

## Abstract

High levels of hepcidin, the main regulator of systemic iron metabolism, lead to various diseases. Targeting hepcidin and lowering its concentration is a possible form of intervention in order to treat these diseases. High turnover rate of hepcidin is a major drawback of therapies directly targeting this peptide. We developed two monoclonal antibodies ABT-207 and h5F9-AM8 which inhibit hemojuvelin/repulsive guidance molecule C (RGMc) and downregulate hepcidin. We conducted single-application and dose response studies to understand the antibodies’ mechanism and subchronic toxicology studies to exclude safety-related concerns. Investigation was carried out at different biological levels through qPCR, Affymetrix, liquid chromatography coupled with mass spectrometry (LC-MS/MS), histopathology, serum iron, unsaturated iron binding capacity (UIBC), and drug concentration measurements. After a single application of these antibodies, hepcidin expression in liver and its serum protein levels were reduced. Serum iron increased for several weeks. The RGMc antibodies show a pronounced dose response relationship in rats with h5F9-AM8 having an IC_50_ (UIBC) of approximately 80-fold higher than ABT-207. When hepcidin levels were downregulated, iron deposition in the liver was visible histologically 1 week post application. These antibody-mediated iron depositions were not associated with any adverse toxicologically relevant effect at the doses and time points evaluated. Iron depositions seen after 14 weekly treatments with ABT-207 were reversible in rats and in cynomolgus monkeys. Due to their long-lasting effects and excellent safety profile, both RGMc-blocking antibodies ABT-207 and h5F9-AM8 are favorable clinical candidates for diseases characterized by high serum hepcidin levels like anemia of chronic disease.

**Electronic supplementary material:**

The online version of this article (doi:10.1208/s12248-015-9770-4) contains supplementary material, which is available to authorized users.

## INTRODUCTION

Anemia of chronic disease (ACD), also known as anemia of inflammation, is the most common anemia in hospitalized patients (1). ACD’s pathogenesis starts with an inflammatory response which is accompanied by inflammatory cytokine release mediating disease progression. The cytokines reduce production of erythrocytes, facilitate lysis of erythrocytes, and stimulate macrophages to store and retain iron as ferritin which ultimately leads to insufficient iron availability (2). In addition, interleukin-6 stimulates hepatic hepcidin expression and hepcidin in turn induces degradation of ferroportin thereby blocking iron release from macrophages and enterocytes into the circulation (3).

Hepcidin is the master regulator of systemic iron metabolism. It is synthesized in the liver and the synthesis is controlled by repulsive guidance molecule C (RGMc), a glycosyl-phosphatidylinositol (GPI)-linked glycoprotein (aka hemojuvelin) (4). RGMc has been shown to bind to neogenin, an ubiquitously expressed transmembrane protein with numerous functions (5), and to bone morphogenetic protein 6 (BMP6). RGMc- and neogenin-deficient mice show a decreased BMP signaling pathway and as a consequence reduced liver hepcidin expression (6, 7) suggesting that they jointly regulated the BMP/Smad-mediated signaling pathway of hepcidin regulation (8).

Due to the increased serum hepcidin levels in ACD, many strategies target hepcidin to reduce its serum levels to prevent ferroportin degradation (9, 10). This enables ferroportin-induced iron to be exported out of the cell. Free iron released by ferroportin will be bound to transferrin in the serum which would then be accessible to consumer cells. Due to hepcidin’s high turnover rate with an estimated production rate of 7.6 nmol kg^−1^ h^−1^ and a half-life of 2.3 min in cynomolgus monkeys (11), inhibition of hepcidin can only be achieved by sustaining high dose levels or frequent dosing of anti-hepcidin antibodies.

ABT-207 and h5F9-AM8 are humanized monoclonal antibodies (mAbs) developed at AbbVie. As previously reported, these mAbs possess mid (ABT-207) and high (h5F9-AM8) binding affinity towards repulsive guidance molecule A (RGMa) (Demicheva *et al*. in press Cell Reports 2015) and RGMc. RGMa shares 47% amino acid identity with RGMc (12) and RGMc is conserved in rat, in monkey, and in human (13). Since the rat RGMc protein is responsive to a humanized antibody, most of the animal work was conducted in rats.

Here, we describe these RGMa/c antibodies in detail. RGMc is a crucial constituent of the neogenin-BMP6-BMP receptor complex most important for regulating hepcidin expression. Since both antibodies have long-lasting effects on hepcidin expression, the challenge of high hepcidin turnover rates could be overcome, making these antibodies suitable clinical candidates for the treatment of ACD.

In the first step to characterize these antibodies, we carried out single-dose studies with ABT-207 and h5F9-AM8 in rats to understand time-course and duration of effects on iron metabolism. These studies were followed by dose response studies to establish the relationship between the dose of mAbs and the monitored iron metabolism effect. Since these antibodies are potential clinical drug candidates, hepatic microarray analysis and toxicology studies in two species were performed to analyze safety-related concerns. With all these studies, we could determine the no effect level (NOEL) for both mAbs and we could also demonstrate that both these mAbs are well tolerated. In addition, *in vitro* and *in vivo* pharmacokinetics and pharmacodynamics (PK/PD) relationship between ABT-207 and h5F9-AM8 could be established.

## METHODS

### Generation of ABT-207 and h5F9-AM8

ABT-207 is a monoclonal antibody (mAb) humanized from a rat hybridoma mAb 5F9. h5F9-AM8 is an antibody affinity-matured from ABT-207 by yeast surface display. Both ABT-207 and h5F9-AM8 bind to human, cynomolgus monkeys, rat, and mouse RGMc. They also cross-react with RGMa, another member of the RGM family. However, the observed effect on hepcidin and iron metabolism is associated with RGMc but not RGMa, since an RGMa-specific mAb with no RGMc cross-reactivity failed to show any effect on iron metabolism (data not shown). There was no cross-reaction with other non-RGM molecules observed (e.g., and tissue cross-reactivity with a wide panel of human tissues). The affinity difference between human and cynomolgus monkey RGMc could be due to the different sequences in the binding epitopes of ABT-207 between these two species.

### Animal Studies

Single-dose studies were carried out by dosing 200 mg/kg ABT-207 and 20 mg/kg h5F9-AM8 or vehicle intravenously into 8-week-old female Sprague Dawley (SD) rats. Necropsy was carried out at 4, 8, 24, 48, and 96 h and 1, 2, 3, 4, 5, 6, 7, 8, 9, 10, 11, and 12 weeks post injection (*n* = 5/group). In the dose response studies, 8-week-old female SD rats were injected with 1, 5, 10, and 60 mg/kg ABT-207 or 0.02, 0.2, 2, and 20 mg/kg h5F9-AM8 intravenously (*n* = 5/dose/group) with four weekly doses. For the subchronic toxicology studies with rats and monkeys, ABT-207 was given weekly (total, 14 times) to 8-week-old SD rats at 2, 8, 40, and 200 mg/kg (*n* = 15/sex/group including 5 recovery animals/gender/group) and to 2–5-year-old cynomolgus monkeys at 2, 8, 40, and 160 mg/kg per dose (*n* = 6/sex/group including 2 recovery animals/sex/group). Additional animals from mid- and high-dose groups were kept for a 12-week recovery phase. Detailed study designs can be found in the supplement data.

### Drug Concentration Analysis

This analysis was carried out with Meso Scale Discovery (MSD) immunogenicity assay using serum samples. The detailed protocol can be found in the supplementary section.

### Histology

Hematoxylin and eosin (HE) staining and iron staining using Perl’s Prussian blue (PPB) staining method on paraffin tissue sections were carried out using manuals adapted from Bancroft JD (14).

### Morphometrics

The morphometric analysis was carried out using Definiens Architect software (Definiens) and analysis was adapted according to Hall *et al*. (15).

### Hematology and Serum Iron and UIBC

Iron parameters (total iron and unsaturated iron binding capacity (UIBC) (Roche Diagnostics)) in serum were analyzed using Roche’s chemistry analyzer cobas c501 according to the manufacturer’s instructions. The blood parameters were analyzed using Siemen’s ADVIA 2120.

### RT-PCR

Two milligrams of frozen liver tissue was reverse transcribed and TaqMan real-time PCR was carried out for hepcidin as previously described (16). The rat assays that were used were hepcidin (Rn00584987_m1, Applied Biosystems) and beta-glucuronidase (Rn00566655_m1, Applied Biosystems) with FAM-labeled probe detection.

### LC-MS/MS Analysis

Hepcidin in serum was analyzed with protein precipitation extraction and liquid chromatography coupled with mass spectrometry (LC-MS/MS). The sample volume was 50 μL and the assay dynamic range was 0.5 to 250 ng/mL. Detailed protocol can be found in the supplementary methods.

### RNA Preparation and Gene Array Analysis

RNA was prepared (*n* = 3) (17) and microarray analysis was performed using the standard protocol provided by Affymetrix, Inc. (Santa Clara) and as previously described (17). The array was then scanned using the GeneChip Scanner 3000 (Affymetrix). The microarray scanned image and intensity files (.cel files) were imported into Rosetta Resolver gene expression analysis software, version 7.2 (Rosetta Inpharmatics), and analyzed for gene expression changes *versus* vehicle control rat livers. The data discussed in this publication have been deposited in NCBI’s Gene Expression Omnibus (18) and are accessible through GEO Series accession number GSE63200 (http://www.ncbi.nlm.nih.gov/geo/query/acc.cgi?acc=GSE63200).

### Statistics

Experimental data from each study were tested for normality using Kolmogorov-Smirnov test and variance homogeneity using Levene’s test and transformed into logarithm scale if needed. Analyses were assessed by one-way analysis of variances followed by Dunnett’s post-hoc test. Statistical analyses were carried out using Graph Pad Prism 5 (GraphPad Software, Inc.) and JMP 10.0 (SAS Institute) software.

## RESULTS

### Single-Dose mAbs Effect on Iron Regulation

**Fig. 1:**
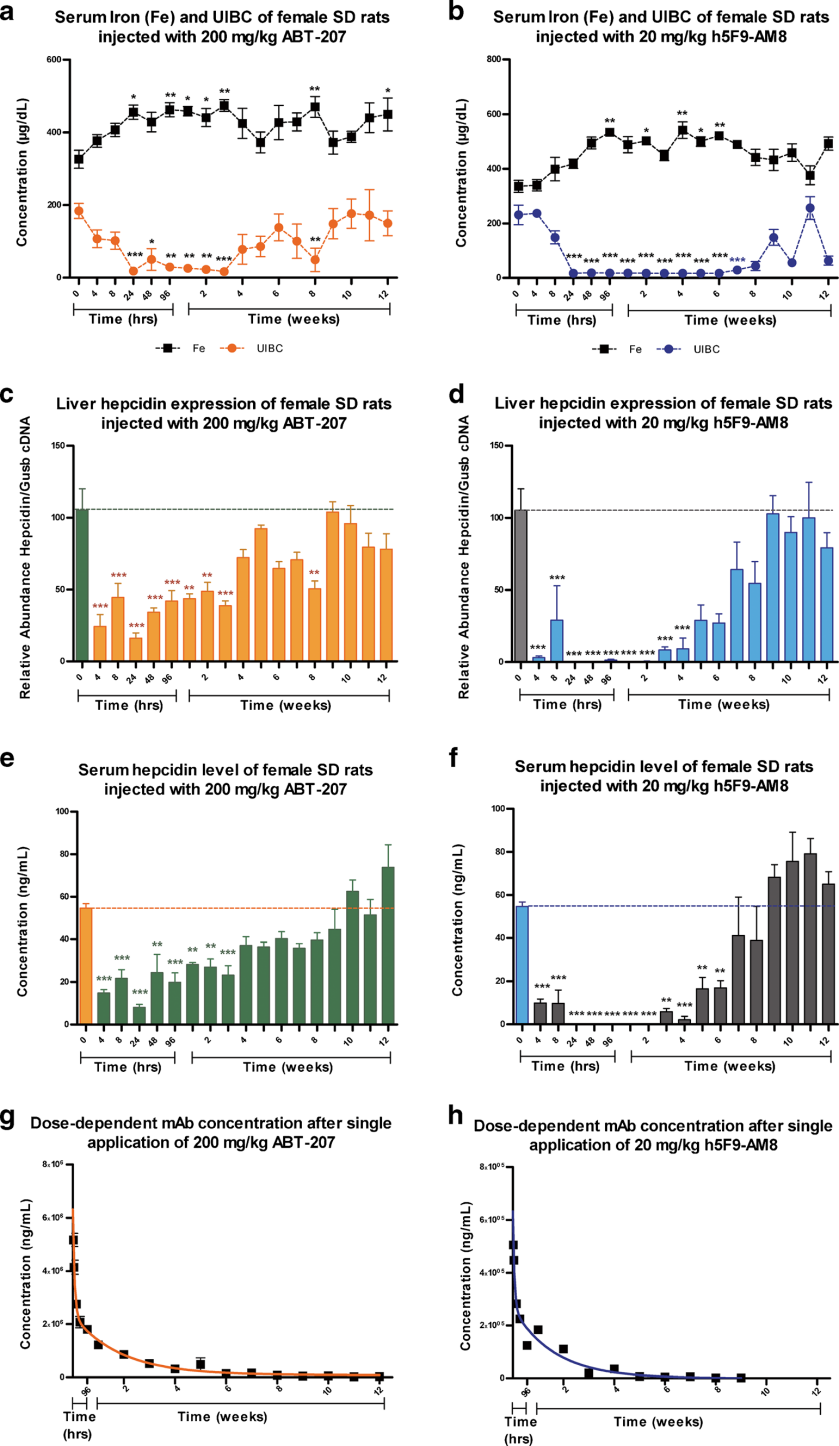
Serum iron, serum UIBC, liver hepcidin expression (mRNA), serum hepcidin level (protein), and antibody concentration in female SD rats after a single 200 mg/kg ABT-207 and 20 mg/kg h5F9-AM8 application. Both mAbs were injected intravenously on day 0 and timed necropsy of animals was carried out at different time points. Parameters measured from rats injected with ABT-207 (*left diagrams*) and h5F9-AM8 (*right diagrams*) include serum iron and UIBC (**a**, **b**), liver hepcidin expression (**c**, **d**), serum hepcidin levels (**e**, **f**), and antibody concentration levels of ABT-207 (**g**) and h5F9-AM8 (**h**) in serum. Statistical analysis shows significant effect (*p < 0.05, **p < 0.01, and ***p < 0.001) from one-way ANOVA conducting Dunnett’s post-hoc test with 0 h group as baseline. Data is displayed in mean and *error bars* represent standard error of the mean (SEM)

In the single-dose studies, no effect on hematology parameters such as the erythrocytes, white blood cells, and hemoglobin due to the administration of ABT-207 (at a single dose of 200 mg/kg) and h5F9-AM8 (at a single dose of 20 mg/kg) antibodies could be detected (data not shown). Total iron and UIBC parameters which were measured in serum of animals treated with ABT-207 and h5F9-AM8 showed an increase in serum iron and a decrease in UIBC post injection. Animals treated with ABT-207 showed a significant (*p* < 0.05) increase in serum iron and a strong decrease in UIBC up to week 3, and the UIBC level in animals treated with h5F9-AM8 stayed below the level of detection until week 6 (Fig. 1a, b).

At the liver messenger RNA (mRNA) level, significant hepcidin downregulation was observed in animals dosed with ABT-207 and h5F9-AM8 until week 3 and week 4, respectively. However, a complete downregulation of hepcidin could only be observed in animals treated with h5F9-AM8 (Fig. 1c, d). Similarly, serum hepcidin levels in animals treated with ABT-207 dropped significantly until week 3, and in animals treated with h5F9-AM8, hepcidin levels were below the level of quantification from 24 h to week 2 post application and were significantly decreased at least for another 4 weeks until week 6 post application (Fig. 1e, f). Serum antibody concentration was also measured in serum of all animals involved in both studies. The volume of distribution for ABT-207 and h5F9-AM8 are 88.34 and 62.20 mg/kg and the clearance of both mAbs are 0.22 and 0.25 mL/h/kg, respectively. The half-life of ABT-207 in rat is approximately 11.7 days and of h5F9-AM8 is approximately 7.2 days (Fig. 1g, h and the enlarged scale of 0–96 h in Supplementary Figure 1).

During necropsy, one part of liver and spleen tissue were directly fixed in formalin and HE and PPB staining were carried out. All the stained slides were analyzed by an experienced pathologist. Based on the HE staining, changes in iron deposition but no other changes in morphology were observed in the examined tissue (data not shown). The semi-quantitative examination of the iron deposition based on PPB staining was compared to the quantitative method using semi-automated morphometric analysis. In the ABT-207-treated liver, iron deposition was seen between week 1 and week 9 and no evident decrease in spleen iron content was seen. The semi-automated morphometric analysis supported the liver iron content findings; however, at the same time, it showed a decrease in the spleen iron content after application (Supplementary Figure 2A).

**Fig. 2:**
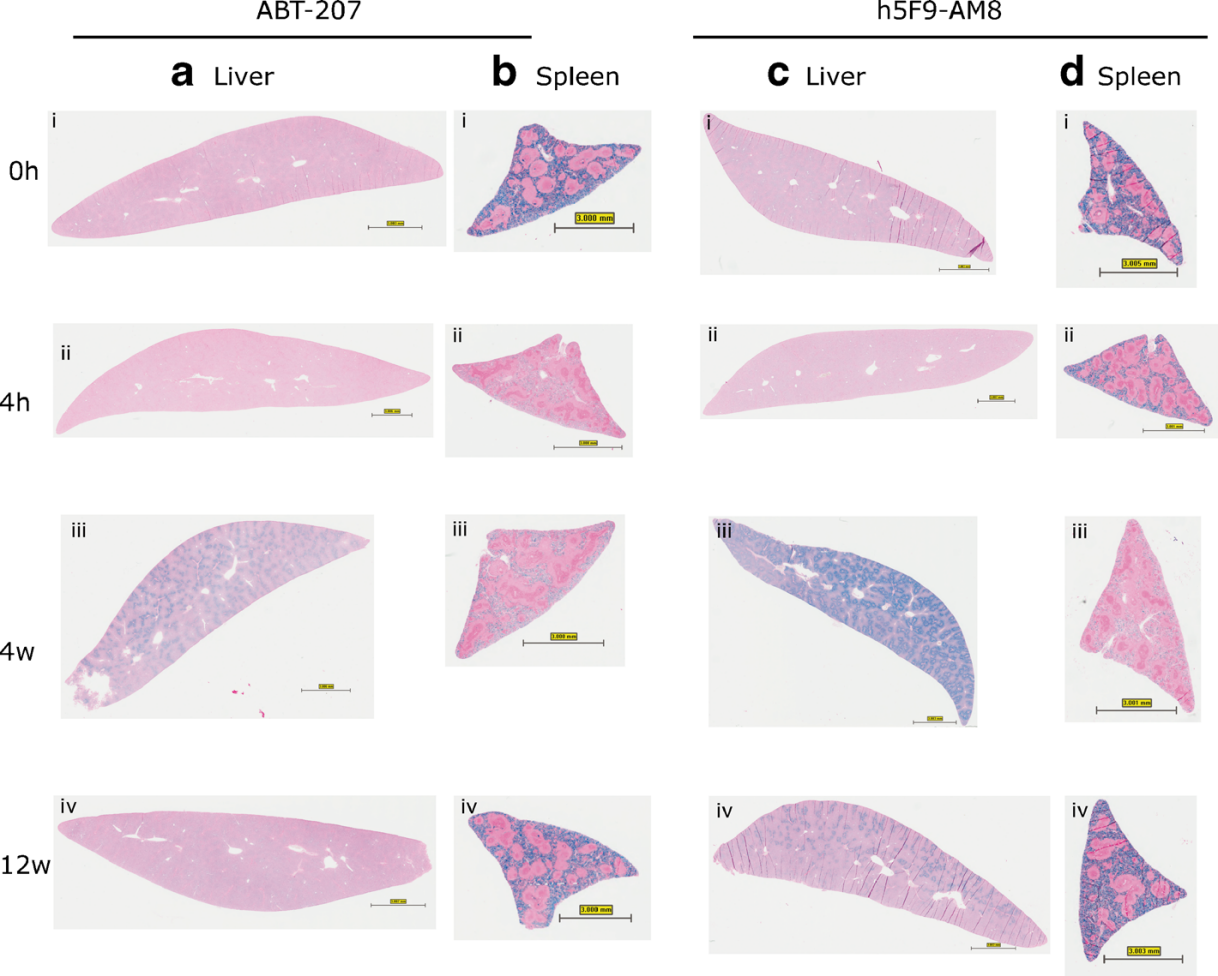
Perl’s Prussian blue staining on liver and spleen sections of rats dosed with ABT-207 or h5F9-AM8. In animals treated with ABT-207, liver iron deposition (**a**) was visible between week 1 and week 9 and iron reduction (**b**) in the spleen could be seen within this time frame. In animals treated with h5F9-AM8 (**c**, **d**), this observation is more pronounced. *Bar* represents 3 mm for all panels

The pattern of an increase of liver iron and decrease in spleen iron content was more evident in the analysis of the h5F9-AM8-treated animals. Liver iron content increased at week 1 and the iron deposition was apparent until week 12 (end of study). In the spleen, iron decrease was seen between week 1 and week 9 (Fig. 2). The semi-automated morphometric analysis for this study correlated well to the spleen iron finding, and this analysis also showed that the liver iron content was decreasing after week 7. An intercept between liver iron’s increase and spleen’s iron decrease was spotted at week 10 (Supplementary Figure 2B).

### Dose Response of mAbs in Selective Iron Regulation-Related Parameters

**Fig. 3:**
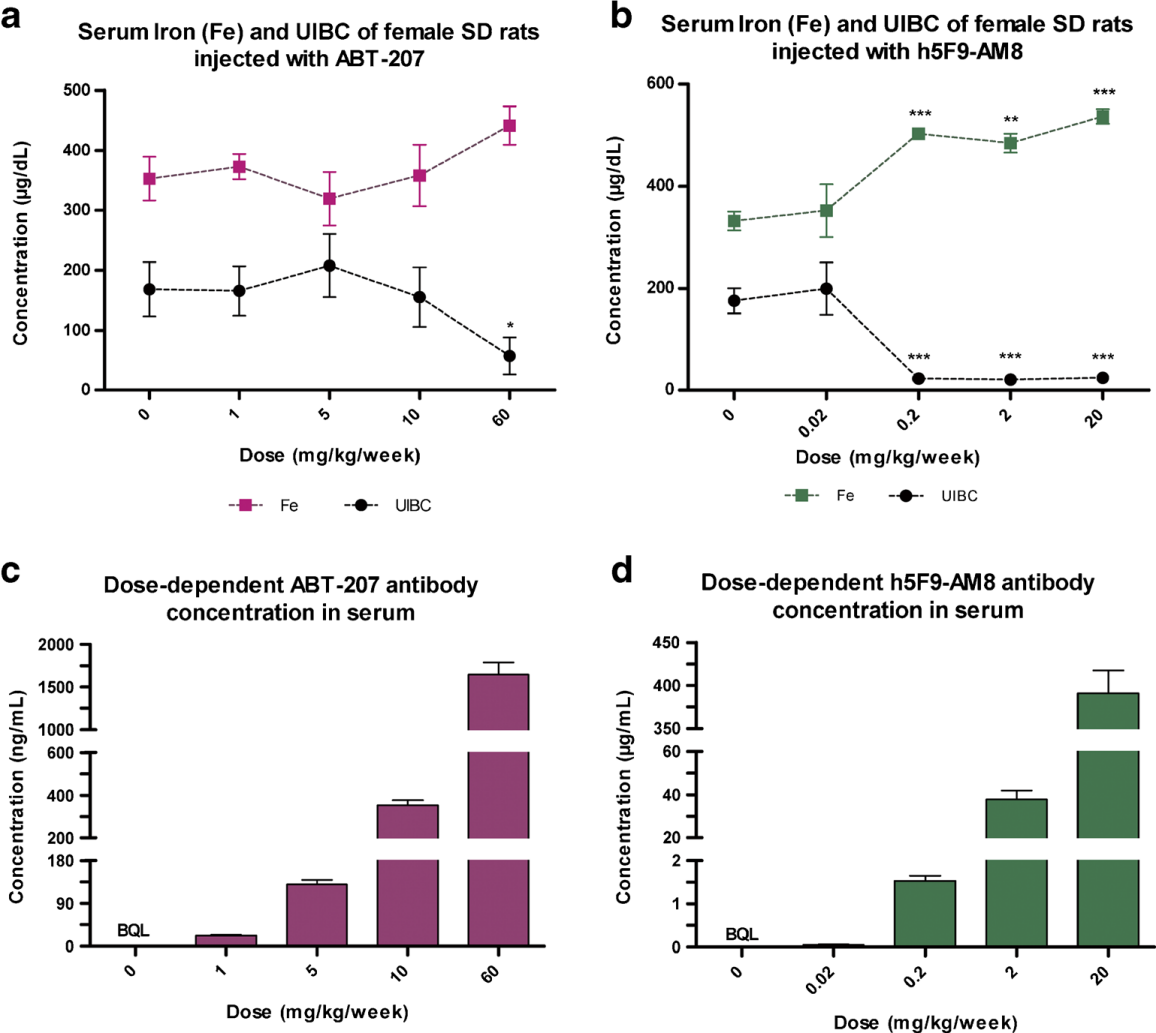
Serum iron, UIBC, and antibody concentration in female SD rats after multiple dosing. ABT-207, h5F9-AM8, and vehicle were applied once weekly for 4 weeks and animals were necropsied 24 h after final dosing. Serum iron levels and UIBC in ABT-207 and h5F9-AM8-treated rats (**a**, **b**). Antibody drug concentration levels of ABT-207 and h5F9.AM8 (**c**, **d**). Statistical analysis in Fig. 3a, b shows significant effect (*p < 0.05, **p < 0.01, and ***p < 0.001) from one-way ANOVA conducting Dunnett’s post-hoc test with 0 mg/kg/week group (vehicle) as baseline. Data is displayed in mean and *error bars* represent standard error of the mean (SEM)

In order to find a relationship between the dose of the mAbs and the effect on hepcidin and iron, ABT-207 and h5F9-AM8 were applied at different doses once weekly for a total of four doses. Increased serum iron levels for animals treated with ABT-207 were observed at 10 and 60 mg/kg/week, and the UIBC level decreased at the same doses (UIBC effect significant at 60 mg/kg/week) (Fig. 3a). In animals treated with h5F9-AM8, a significant increase (*p* < 0.01) in serum iron and a decrease in UIBC level was observed starting at a dose of 0.2 mg/kg/week and at higher doses (Fig. 3b). As expected, the antibody concentrations in serum in both studies were dose related (Fig. 3c, d).

**Fig. 4:**
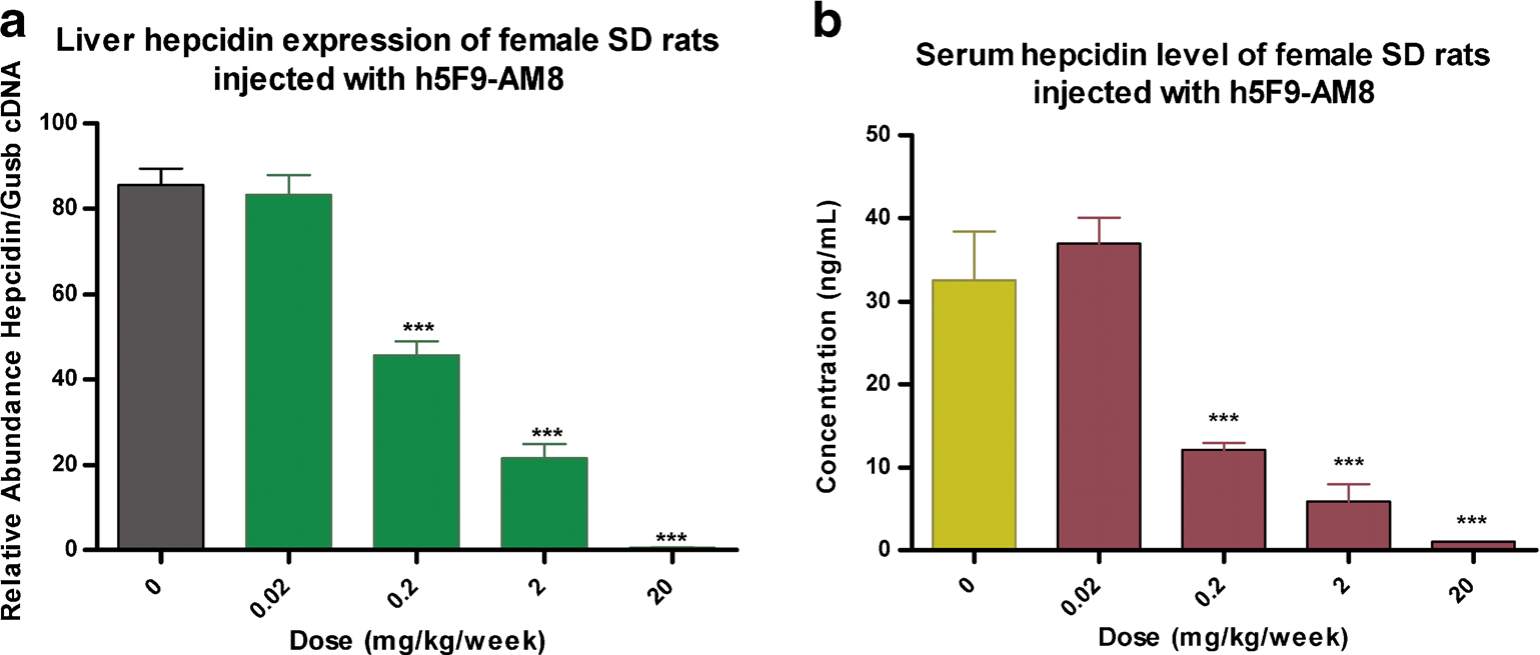
Liver hepcidin mRNA expression and serum hepcidin levels in female SD rats treated with multiple doses of h5F9-AM8. Liver hepcidin expression (**a**) and serum hepcidin level (**b**) showed dose-dependent decreases of hepcidin at doses higher than 0.2 mg/kg/week and no differences between vehicle and 0.02 mg/kg. Statistical analysis shows significant effect (*p < 0.05, **p < 0.01, and ***p < 0.001) from one-way ANOVA conducting Dunnett’s post-hoc test with 0 mg/kg/week group (vehicle) as baseline. Data is displayed in mean and *error bars* represent standard error of the mean (SEM)

In addition, hepcidin mRNA expression in liver and serum hepcidin levels were determined for h5F9-AM8. For both parameters, no differences were seen between vehicle group (0 mg/kg/week) and 0.02 mg/kg/week. However, liver hepcidin mRNA expression and serum hepcidin level decreased proportionally with the dose starting from the minimal effective dose of 0.2 mg/kg/week (Fig. 4a, b). At 20 mg/kg/week, the serum hepcidin level was below the detection limit (Fig. 4b).

**Fig. 5:**
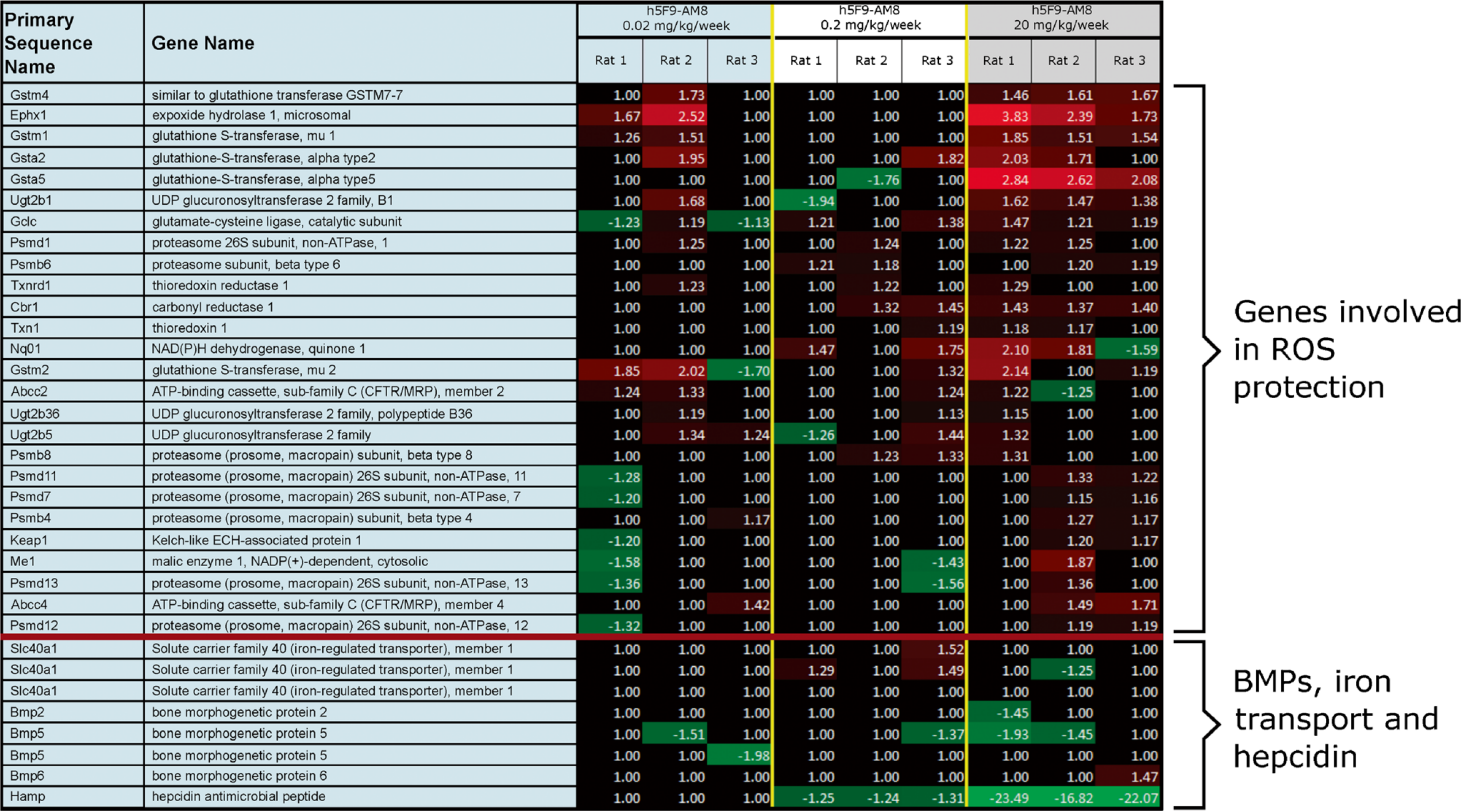
Whole genome transcriptomic profiling with native liver tissue from animals dosed with h5F9-AM8. Comparison between liver tissue from animals at NOEL (0.02 mg/kg/week) with 0.2 and 20 mg/kg/week is shown. Three rats per dose group (0.02, 0.2, and 20 mg/kg) were randomly selected for gene expression analysis. A minimal upregulation of response to oxidative stress genes (*p* ≤ 0.05) was apparent only at 20 mg/kg dose. Hepcidin showed a robust downregulation at the 20 mg/kg dose. *Shades of green* indicate a transcript was downregulated, *shades of red* indicate upregulation, and *black* indicates no significant change (*numbers* indicate fold change relative to vehicle livers)

To elucidate the antibody effect on genes other than hepcidin, a whole genome transcriptomic profiling experiment with liver tissue samples from this study was conducted. The results showed that h5F9-AM8 strongly downregulated hepcidin mRNA expression approximately 20-fold. Some minor upregulation (2–3-fold) for genes involved in oxidative stress protection (mostly at the 20 mg/kg dose) and minor perturbations for select BMPs and ferroportin (Slc40a1) were also observed (Fig. 5). Globally, a minimal number of hepatic gene expression changes were observed (1023 probe sets with a fold change ≥ ±2.0 and *p* ≤ 0.01 for at least one rat, corresponding to approximately 3.3% of the array).

### Safety Assessment of ABT-207

**Fig. 6:**
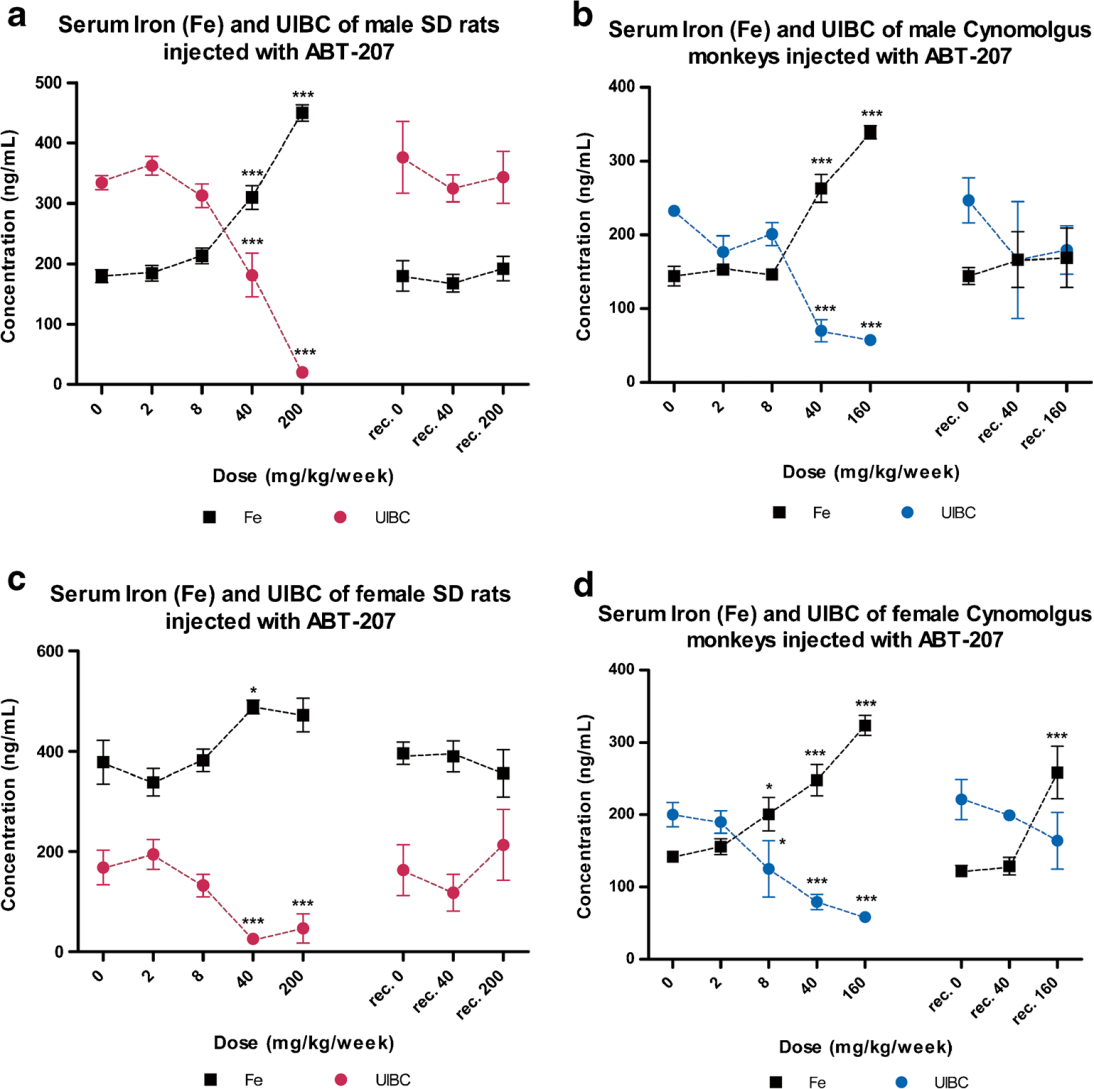
Serum iron and UIBC of SD rats and cynomolgus monkeys treated with 14 doses of ABT-207 once weekly followed by a 12-week recovery period. SD rats and cynomolgus monkeys were treated with ABT-207 at doses of 0, 2, 8, 40, and 200 mg/kg (rats) and 0, 2, 8, 40, and 160 mg/kg (cynomolgus monkeys) for 14 weeks and subsequently kept under recovery phase for 12 weeks. Total recovery of serum iron and UIBC was seen after 12 weeks for rats (**a**, **c**) and cynomolgus monkeys (**b**, **d**). Statistical analysis shows significant effect (*p < 0.05, **p < 0.01, and ***p < 0.001) from one-way ANOVA conducting Dunnett’s post-hoc test with 0 mg/kg/week group (vehicle) as baseline. Data is displayed in mean and *error bars* represent standard error of the mean (SEM)

The toxicological assessment of ABT-207 was done for both males and females in rodents and in non-human primates. Both rats and cynomolgus monkeys were treated once weekly for 14 weeks with ABT-207 at four different doses and kept in the recovery phase for 12 weeks. In the rats, an increase in serum iron and a decrease in UIBC for both males and females and a gender difference could be observed. The males were more sensitive during the dosing period in this study since a pronounced effect in serum iron and UIBC could be seen between 40 and 200 mg/kg/week dose. However, both sexes showed normalized blood parameters after the recovery period (Fig. 6a, c).

The observation of the iron parameters in ABT-207-treated cynomolgus male and female monkeys during the dosing period was similar. An increase in serum iron and a decrease in UIBC level could be seen at doses of 40 and 160 mg/kg/week in male and female cynomolgus monkeys, and a total recovery could be seen after 12 weeks (Fig. 6b, d).

Evaluation of the PPB-stained tissue sections also revealed that the iron effect, present in mid- and high-dose groups, was not adverse and was partly reversible after the recovery phase (data not shown).

## DISCUSSION

Iron-restricted medical disorders caused by excessive hepcidin, e.g., in ACD, iron-refractory iron deficiency anemia (IRIDA), or anemia of chronic kidney disease, are lacking suitable therapeutic options, and the current treatment strategies are accompanied by undesired side effects (1). All current approaches targeting hepcidin and bone morphogenetic protein receptor (BMPR) directly may have major drawbacks due to hepcidin’s high turnover rate and BMPR’s involvement in many other cellular processes (19,20).

ABT-207 and the affinity-matured h5F9-AM8 mAb are mAbs targeting repulsive guidance molecule (RGM) with specific binding affinity towards RGMa and RGMc, and this binding also inhibits the RGMc-BMP2/4/6 interaction. These antibodies differ in their binding affinity towards RGMa and RGMc (ABT-207 rat *K*_D_ 59 nM and h5F9-AM8 rat *K*_D_ 0.24 nM) (Supplementary Table 1). A cellular reporter gene assay, specific for RGMc-BMP2/4, showed that IC_50_ for these antibodies differed approximately 50-fold between ABT-207 (IC_50_ 17 nM) and h5F9-AM8 (IC_50_ 0.37 nM) (Kovac *et al*. submitted—under review). These data indicate that affinity maturation led to a more efficacious inhibition of the RGMc-BMP-driven effects. Since it is known that BMPs are deeply involved in iron metabolism pathway, we decided to investigate *in vivo* to which extent ABT-207 and h5F9-AM8 are involved in iron regulation.

As ABT-207 and h5F9-AM8 antibodies showed different efficacy *in vitro*, single-application studies were carried out for both antibodies. When we administrated 200 mg/kg ABT-207 or 20 mg/kg h5F9-AM8 to rats, we detected an increase in serum iron and a decrease in UIBC (Fig. 1a, b). With ABT-207, these differences were significant until week 3 post injection whereas treatment with h5F9-AM8 showed a higher magnitude and significantly longer-lasting effects up to six weeks. Liver hepcidin mRNA expression was significantly reduced for 8 weeks in ABT-207- and h5F9-AM8-treated animals; however, a more pronounced effect was seen for h5F9-AM8. Correlating to the data on mRNA level, the serum hepcidin protein concentration was suppressed significantly after ABT-207 application for 3 weeks and up to 6 weeks after h5F9-AM8 (Fig. 1c–f).

Since our antibodies bind to RGMc and block the RGMc-BMP interaction which induces downregulation of liver-derived hormone hepcidin, it is very likely that iron release through ferroportin is the intermediate step in this pathway. The data supports the abovementioned notion that these mAbs specifically act via the BMP-hepcidin-ferroportin pathway albeit with different efficacy. Xiao *et al*. reported that hepcidin has high turnover rate (approximately 2.3 min in cynomolgus monkeys) (11), but the binding of our antibodies to RGMc and not directly to hepcidin solved this issue. Due to the antibodies’ long half-lives in rats (7.2–11.7 days), hepcidin was successfully downregulated for several weeks and this negatively correlates with the serum iron levels. The fading antibody effect was only seen after several half-lives when the antibody concentration is extremely low.

In order to evaluate the dose response relationship for ABT-207 and h5F9-AM8, dose escalation studies were conducted in rats. Antibody concentrations measured in serum samples of all animals were dose related. In rats dosed with ABT-207, an increase in serum iron and a decrease in UIBC were seen at a dose of 60 mg/kg/week (NOEL = 10 mg/kg). On the other hand, in rats dosed with h5F9-AM8, these changes were already observed at a minimal dose of 0.2 mg/kg/week (NOEL = 0.02 mg/kg) (Fig. 3). For both antibodies, a dose response relationship could be established for the UIBC. ABT-207 had an IC_50_ of 12.74 mg/kg and h5F9-AM8’s IC_50_ was 0.15 mg/kg, which had approximately 80-fold difference for this parameter (Supplementary Figure 3A and Supplementary Figure 4A).

With this result, we could establish a good correlation between the *in vivo* and *in vitro* data (Kovac *et al*. in preparation). Since both antibodies have comparable pharmacokinetic parameters, the differences in efficacy *in vivo* are most likely driven by the differences in their binding affinities.

In order to investigate the specificity of the antibody effect on hepcidin expression, we chose the affinity-maturated h5F9-AM8 antibody. A whole genome transcriptomic profiling (Affymetrix) experiment was conducted. There were a minimal number of global gene expression changes for the NOEL (0.02 mg/kg), mid dose (2 mg/kg) and the highest dose (20 mg/kg). The most downregulated gene in the dataset was hepcidin, and only minor modulations were apparent with respect to select BMPs and ferroportin (Slc40a1) (Fig. 5).

Based on this analysis, we conclude that h5F9-AM8 only induces iron effects and only minor perturbations on the liver. Potential toxicological consequences of excess iron may include production of free radicals and other reactive oxygen species. Gene expression signals indicate a minor induction of an oxidative stress response only at the 20 mg/kg dose, including increased GSTs and proteasome subunits. In order to quantify the dose response effect of h5F9-AM8, we analyzed hepcidin mRNA expression, serum hepcidin levels, liver iron deposition, and spleen iron content. Liver hepcidin mRNA expression and serum hepcidin level analyses revealed a closely related dose-dependent decrease for both parameters (Fig. 4). The IC_50_ for liver hepcidin was determined to be approximately 0.08 and 0.03 mg/kg for serum hepcidin (Supplementary Figure 3B-C).

Under some pathological conditions (hemochromatosis), low hepcidin levels result in increased serum iron, periportal iron accumulation in liver, and spleen iron depletion; we asked if this condition occurs due to the application of ABT-207 and h5F9-AM8 as well. Single-application studies with these antibodies showed periportal liver iron deposition between week 1 and week 9 in animals treated with 200 mg/kg ABT-207 and between week 1 and week 12 (end of study) in animals treated with 20 mg/kg h5F9-AM8. We also observed reduction of iron load of macrophages in the spleen within this time frame (Fig. 2). The PPB-stained liver and spleen tissue sections were quantified with a semi-automated morphometric analysis (Supplementary Figure 2). This analysis supported the qualitative histological findings confirming the stronger effect of h5F9-AM8 in comparison to ABT-207. Also in the dose response studies, a dose-dependent iron accumulation in the liver and iron depletion in the spleen was observed.

In order to access possible toxicological findings and reversibility of the effects generated, we conducted 13-week (14 weekly injections of ABT-207) toxicology studies followed by a 12-week recovery period in rats and cynomolgus monkeys. Besides the usual assessment of toxicology parameters, iron metabolism was monitored through serum iron, UIBC, and PPB staining (data not shown). Through the toxicology studies, we could demonstrate that rats were the more sensitive species (UIBC IC_50_ 12.74 mg/kg/week) compared to the cynomolgus monkeys (UIBC IC_50_ 34.73 mg/kg/week) (Supplementary Figure 4). The only finding induced by ABT-207 in these studies was a dose-dependent effect of ABT-207 on iron parameters which were assessed as non-adverse and showed reversibility for all serum parameters after the recovery phase.

In addition, all *in vivo* studies did not result in test item-related clinical signs, body weight changes, mortality, or any other adverse findings including morphological changes of all examined tissues. The antibody also had low immunogenic potential (very low evidence for the development of anti-drug antibodies (ADAs)).

## CONCLUSION

In summary, we showed that both ABT-207 and h5F9-AM8 downregulate liver hepcidin production which in consequence alters iron metabolism and disposition. Moreover, this intervention is highly specific since it fits to the RGMc-hepcidin-iron cascade (increase of serum iron, decrease in spleen iron, and liver iron accumulation). No adverse effects including low incidence of ADAs could be detected in all studies conducted so far (0.025% in rats and no incidence in cynomolgus monkeys), and all other serum effects (serum iron and UIBC) were reversible. Iron accumulation in liver was partially reversible. All these outcomes point to a specific intervention on a druggable target and open a new dimension in the treatment of hepcidin-related anemia. ABT-207 and in particular the h5F9-AM8 which acts as a specific and highly efficacious antibody with a relatively long half-life seem suitable for medical indications in which hepcidin upregulation plays a role. Further animal studies using these antibodies, within appropriate disease animal models, are performed and are described (Kovac *et al*. submitted—under review).

## Electronic Supplementary Material

ESM 1 (DOC 20,219 kb)
