# Supplementary material for: Anti-repulsive Guidance Molecule C (RGMc) Antibodies Increases Serum Iron in Rats and Cynomolgus Monkeys by Hepcidin Downregulation
Source: AAPS J. 2015 Apr 22;17(4):930–8. doi: 10.1208/s12248-015-9770-4 (PMC4476998; doi:10.1208/s12248-015-9770-4)
Supplement: Supplementary file 1 — (DOC 20,219 kb) [file 12248_2015_9770_MOESM1_ESM.doc]

**Anti-Repulsive Guidance Molecule C (RGMc) antibodies increases serum iron levels in rats and cynomolgus monkeys by hepcidin regulation**

**Supplementary Methods**

***Animal studies.*** For the single dose study, 8 week old female Sprague Dawley (SD) rats (Charles River) weighing approximately 200g were acclimated for five days and baseline blood parameters were determined from all animals. Single IV application of 200 mg/kg ABT-207, 20 mg/kg h5F9-AM8 or vehicle were given to the rats respectively (n=5/group). Timed necropsy was carried out at 4, 8, 24, 48, 96 hours and 1, 2, 3, 4, 5, 6, 7, 8, 9, 10, 11, 12 weeks post injection. In the dose response study, groups of five female SD rats were injected with 1, 5, 10 and 60 mg/kg ABT-207 or 0.02, 0.2, 2, and 20 mg/kg h5F9-AM8 intravenously and for both studies animals in the control group received vehicle. In both studies antibody was given once weekly (total 4 doses), and animals were necropsied 24 hours after the final dose. The subchronic toxicology studies in rats and cynomolgus monkeys were carried out with 14 weekly applications of 2, 8, 40 and 200 mg/kg ABT-207 in rats and 2, 8, 40 and 160 mg/kg ABT-207 in monkeys followed by a 12 week recovery period of animals in the control, 40 mg/kg and high dose groups. Ten (including five animals for recovery) males and females were used in each main group for the rat study and six (including two animals for recovery) male and female cynomolgus monkeys were used in the non-rodent studies. During necropsy, blood was collected for hematology analysis, for determination of serum iron and UIBC and for the antibody concentration in serum. . Liver and spleen were collected; one part was fixed in 10% buffered formalin and the other part was snap frozen in liquid nitrogen and stored at -80°C for further analysis. All rat studies were carried out according to Abbvie’s animal guidelines and according to the German Animal Welfare Act and European regulations and were approved by the local external authorities. The cynomolgus monkeys studies were carried out according to the U.S. Department of Agriculture’s (USDA) Animal Welfare Act (9 CFR Parts 1, 2 and 3) and were approved by the local IACUC authorities.

***Drug Concentration Analysis.*** Calibration-Curve-Standards (STDs), Quality Controls (QCs) and study samples were diluted in deep well plates (Greiner bio-one) and incubated at 4°C overnight. To conduct the assay, 96-well standard plates Mesoscale Discovery (MSD) were used. First 70 µL 3% Blocker (MSD) A blocking buffer was pipetted per well and incubated for one hour at room temperature. Plates were washed three times with 300 µL of 1x TTBS wash buffer (Rockland) and air dried. Thereafter 25 µL/well of biotinylated RGMa (2 µg/mL; AbbVie) was pipetted and the plates were incubated for one hour at room temperature. Plates were washed again and 70 µL/well of the STDs, QCs and samples were added as duplicates and the plates were incubated for 90 minutes at room temperature. This step was followed by washing and a 90 minute incubation of 70 µL/well (1 µg/mL) of an anti-human Antibody labeled with Ru(bpy)3; (Star97, Serotec). During all incubation steps the standard plates were placed on a MTS-shaker which was set to 600 rpm. The plates were washed again and 150 µL/well of 2x Read Buffer (MSD) was pipetted into each well. The amount of ABT-207 or h5F9-AM8 bound was measured by relative luminescence units (RLU) which is proportional to the RLU.

***LC/MS/MS analysis.*** Rat hepcidin stock solution was prepared by adding 1 mL of Millipore water to the vial containing 54 g rat hepcidin reference material (Peptide Institute Inc.). Standard samples were prepared at 201, 182, 82.7, 28.1, 9.24, 2.48, 1.02 ng/mL and QC samples were prepared at 162, 18.8, and 1.52 ng/mL in charcoal stripped rat serum (BioChemed).

50 µL of thawed rat serum and 150 µL of 0.1% formic acid ACS grade (Fischer Scientific) in water were added to each well of a 2.2 mL square-well extraction plate. The samples were vortexed for 1 minute at 1300 rpm. 50µL of the internal standard (IS) (100 ng/mL isotopic labeled human hepcidin mHepcidin-IS (Peptide Institute Inc., Japan)) in 50/50/0.1 (v/v/v) methanol/water/formic acid was added to the appropriate wells. The blank wells were filled with 50/50/0.1 (v/v/v) methanol/water/formic acid. The samples were mixed by vortexing them for 1 minute at 1300 rpm. 600 µL of 0.1% formic acid in acetonitrile was added to all wells of the extraction plate. The extraction plates were heat sealed and the vortexed for 3 minutes at 1800 rpm and centrifuged for 5 minutes at 3400 rpm at 10°C. 500 µL of the supernatant was transferred into clean 1.2 mL plate. The content was dried under a stream of heated nitrogen (70°C) stream and reconstituted with 100 μL of 50/50/0.1 (v/v/v) methanol/water/formic acid. The plate was covered with a plate mat and vortexed for 1 minute at 1300 rpm. 50 µL of sample was injected into the LC-MS/MS.

A Phenomenex Kinetex C18 (2.6 μm, 2.1 x 50 mm) HPLC column was used for separation. Mobile phase A consisted of 5/95/0.1 (v/v/v) methanol/water/formic acid and mobile phase B consisted of 95/5/0.1 (v/v/v) methanol/water/formic acid. The HPLC was maintained with 10% of mobile phage B from 0 to 1.5 minutes. Between 1.5 to 5 minutes the HPLC was ramped up to a gradient of 10-90% mobile phase B. A constant flowrate of 0.4 mL/min was maintained and 30 µL of the final eluent was injected into a Sciex API5000 triple quadrupole mass spectrometer with a turbo iron spray interface. Spray needle voltage was 3800 V and the source temperature was 650°C. MS/MS transitions that were monitored were 905.0 (+3 state) to 1118.5 m/z for rat hepcidin and 703.4 (+4 state) to 354.1 m/z for mHepcidin-IS. Bias (%) (measured concentration/nominal concentration) and coefficient of variation (CV %) was used to evaluate the reproducibility of the results from all the samples. All data was acquired and processed by Sciex Analyst version 1.6.

**Supplementary Tables**

**Supplementary TABLE 1**

Supplementary Table 1: RGMc binding affinity of ABT-207 and h5F9-AM8.

| **RGMc sample** | **ABT-207** | | **h5F9-AM8** | |
| --- | --- | --- | --- | --- |
|  | **K_D_ (nM)** | **Fit** | **K_D_ (nM)** | **Fit** |
| Human RGMc | 31 | Two State Binding | 0.1 | 1:1 Binding |
| Cyno RGMc | 2.5 | Two State Binding | 0.11 | 1:1 Binding |
| Rat RGMc | 59 | Steady State Affinity | 0.24 | 1:1 Binding |

**Supplementary Figures**

Supplementary FIGURE 1

| 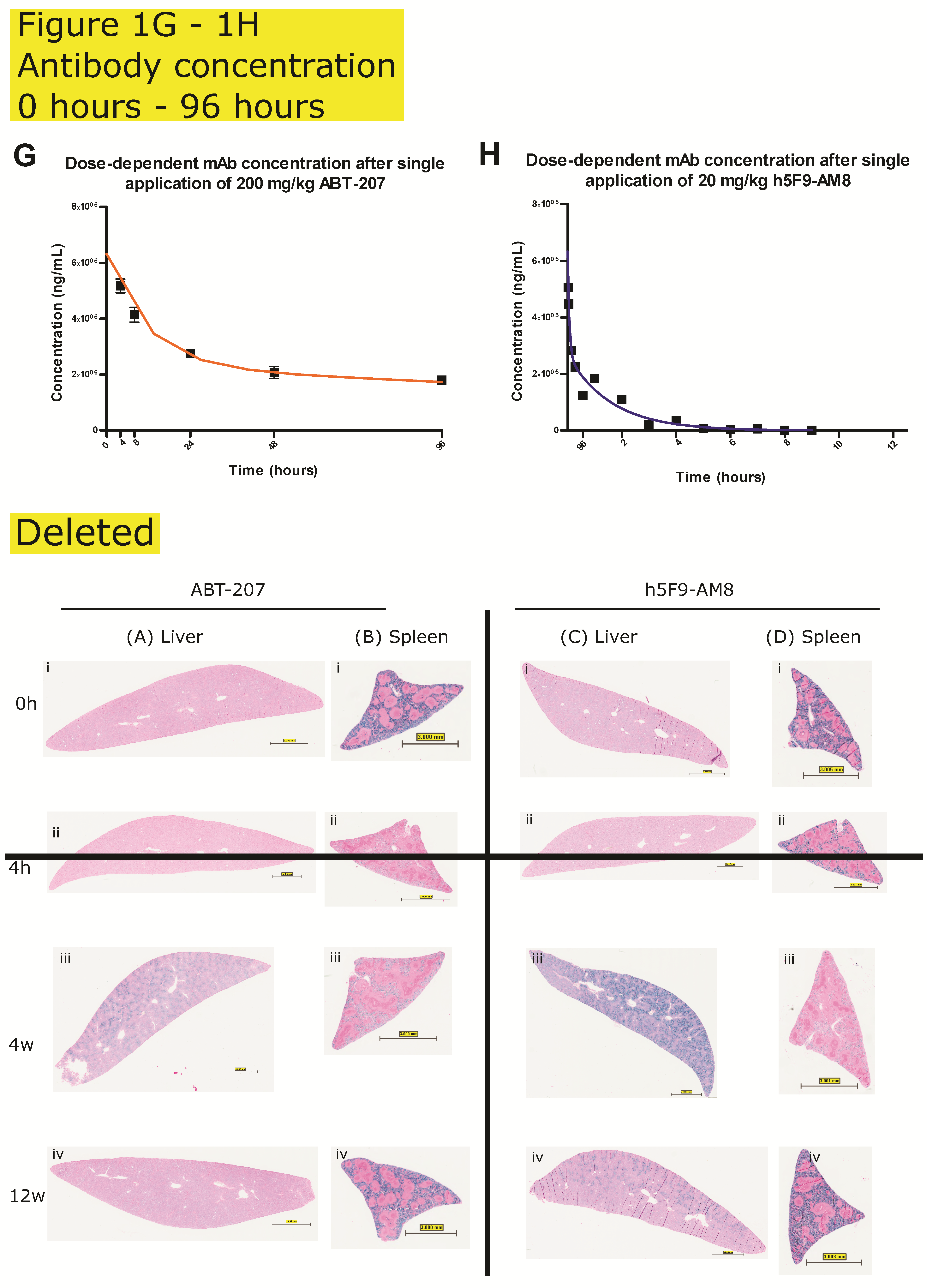 |
| --- |

**Supplementary Figure 1: Antibody concentration between 0-96 hours post single application of 200 mg/kg ABT-207 or 20 mg/kg h5F9-AM8.** (G) Concentration of ABT-207 at 4 hours post application is approximately 5 x 10^6^ ng/mL and approximately 1.8 x 10^6^ ng/mL of ABT-207 was still present in the serum at 96 hours. (H) The concentration of h5F9-AM8 was approximately 5 x 10^5^ ng/mL at 4 hours and approximately 1.2 x 10^5^ ng/mL h5F9-AM8 at 96 hours post application.

**Supplementary FIGURE 2**

**
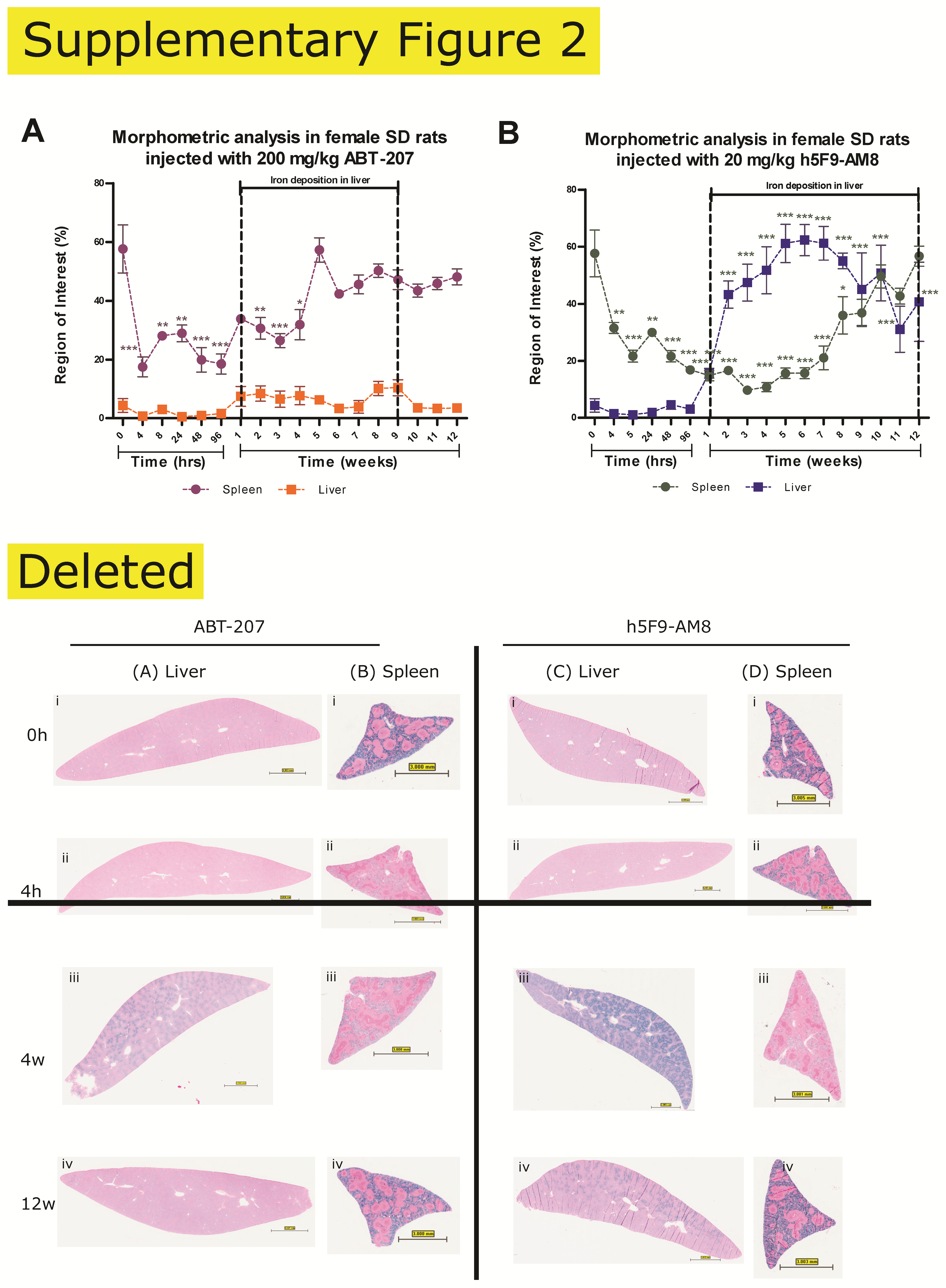
**

**Supplementary Figure 2: Perl’s Prussian blue staining quantification using morphometric analysis**. Iron deposition was determined using PPB and tissue sections were examined by a pathologist. The staining intensity of PPB staining in liver and spleen was quantified using morphometrics and dashed lines indicate the period during which increased iron deposition in the liver was noted by the pathologist. (A) A small increase in iron deposition in the liver of animals treated with ABT-207 (200 mg/kg) was detected between week 1 and week 9. (B) In animals treated with a single dose of 20 mg/kg h5F9-AM8, a strong increase in iron deposition in the liver was observed one week after antibody application and could still be seen at week 12 post mab application. Statistical analysis shows significant effect (*=p<0.05, **=p<0.01 and ***=p<0.001) from one-way ANOVA conducting Dunnett’s post-hoc test with 0 hours group as baseline. Data is displayed in mean and error bars represents Standard Error of the Mean (SEM).

**Supplementary FIGURE 3**


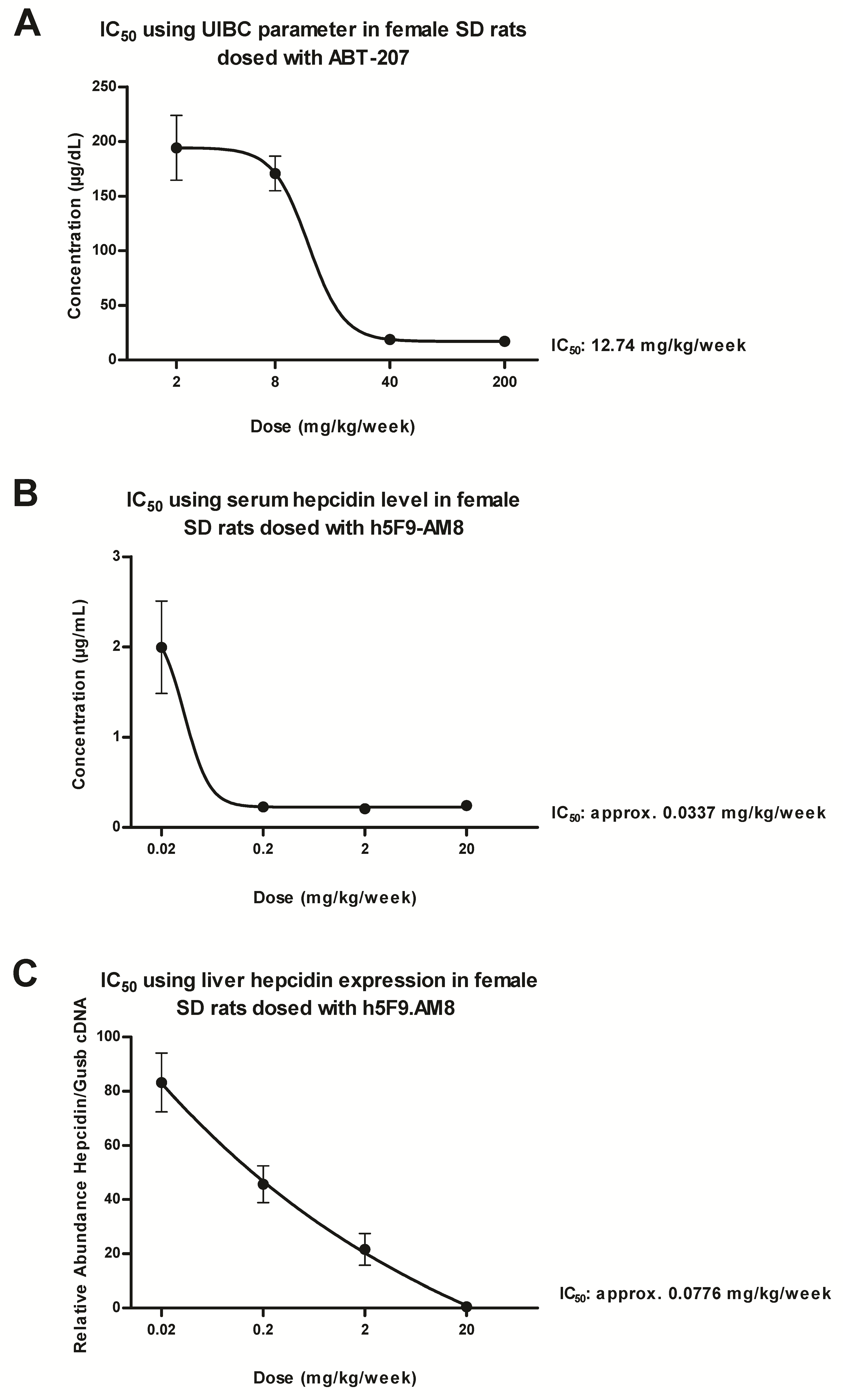


**Supplementary Figure 3: IC_50_ determination for h5F9-AM8 using UIBC, serum hepcidin and liver hepcidin**. (A) IC_50_ of UIBC was approximately 0.1431 mg/kg/week and (B-C) the IC_50_ of serum hepcidin and liver hepcidin were approximately 0.0337 and 0.07765 mg/kg/week.

**Supplementary FIGURE 4**


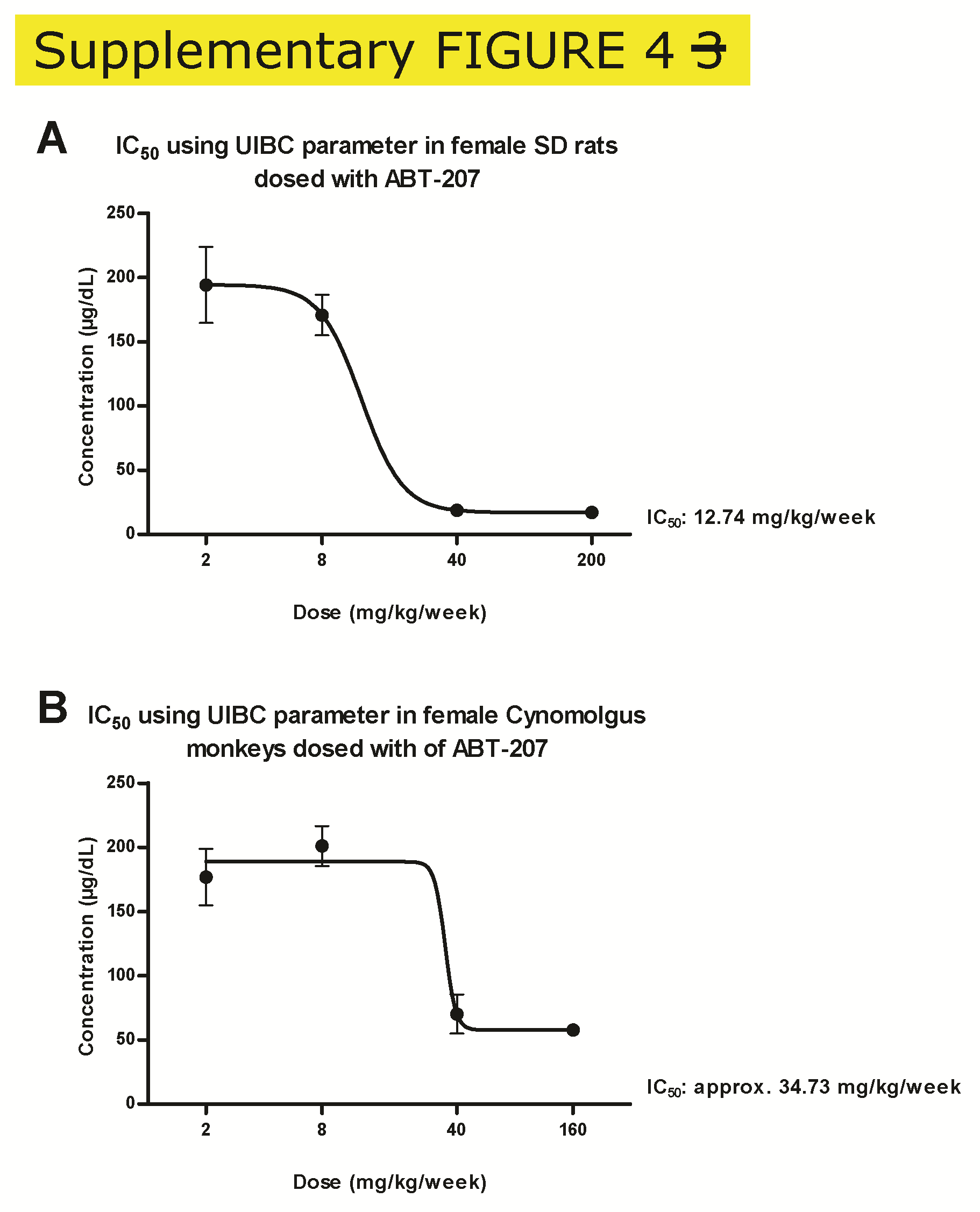


**Supplementary Figure 4: IC_50_ of rats and cynomolgus monkeys dosed weekly for 14 times with ABT-207**. (A) IC_50_ of ABT-207 in rats was 12.74 mg/kg/week and approximately (B) 34.73 mg/kg/week of ABT-207 was needed to achieve the IC_50_ in cynomolgus monkeys.
